# Supplementary material for: Predicting short-term mortality in severe cirrhosis: An interpretable machine learning model integrating routine clinical indicators
Source: PLoS One. 2026 Mar 3;21(3):e0328952. doi: 10.1371/journal.pone.0328952 (PMC12956121; doi:10.1371/journal.pone.0328952)
Supplement: S1 Table — (DOCX) [file pone.0328952.s006.docx]

**S1 Table. Bootstrap test for Cox regression**

| Variable | Mean | Lower CI | Upper CI | HR | HR 95% LCI | HR 95% HCI |
| --- | --- | --- | --- | --- | --- | --- |
| Age | 0.027 | 0.017 | 0.038 | 1.028 | 1.017 | 1.039 |
| AST/ALT | 0.000 | 0.000 | 0.000 | 1.000 | 1.000 | 1.000 |
| INR | 0.106 | 0.053 | 0.191 | 1.112 | 1.055 | 1.211 |
| Creatinine | 0.090 | 0.037 | 0.145 | 1.094 | 1.038 | 1.156 |
| Platelet | -0.004 | -0.006 | -0.003 | 0.996 | 0.994 | 0.997 |
| WBC | 0.050 | 0.039 | 0.068 | 1.051 | 1.040 | 1.070 |
| Bilirubin_total | 0.026 | 0.017 | 0.036 | 1.026 | 1.017 | 1.037 |
| Peptic ulcer disease | -0.609 | -1.187 | -0.126 | 0.544 | 0.305 | 0.881 |

^a^RBC: red blood cell; ^b^ALT: alanine aminotransferase; ^c^ALP: alkaline Phosphatase; ^d^AST: aspartate aminotransferase; ^e^INR: international normalized ratio; ^f^WBC: white blood cell; ^g^BUN: blood urea nitrogen
