# Supplementary material for: Predicting short-term mortality in severe cirrhosis: An interpretable machine learning model integrating routine clinical indicators
Source: PLoS One. 2026 Mar 3;21(3):e0328952. doi: 10.1371/journal.pone.0328952 (PMC12956121; doi:10.1371/journal.pone.0328952)
Supplement: Attachment — Submitted filename: Response to Reviewers.docx [file pone.0328952.s009.docx]

**Response to Reviewers**

**Manuscript ID:** PONE-D-25-36378
**Title:** Predicting Short-Term Mortality in Severe Cirrhosis: An Interpretable Machine Learning Model Integrating Routine Clinical Indicators

**Dear Dr. Sona Frankova and Reviewers,**

We are deeply grateful to you and the reviewers for the thorough and insightful feedback on our manuscript. It has been a privilege to receive such detailed and constructive comments, which have not only strengthened the manuscript considerably but also provided us with valuable guidance for improving the clarity, rigor, and clinical relevance of our work. We have carefully considered each of the points raised and have made extensive revisions to the manuscript to address them. Every suggestion has been taken seriously, and we have implemented changes accordingly to enhance the overall quality of the paper. Below, we provide a detailed, point-by-point response to all the comments. All modifications made in the manuscript have been clearly highlighted in the “Revised Manuscript with Track Changes” file for your convenience.

- **Responses to editor**

**Comment 1：Please ensure that your manuscript meets PLOS ONE's style requirements, including those for file naming.**

**Response 1:** We sincerely thank the editors for their valuable feedback and for providing clear guidance on PLOS One’s style requirements. We have carefully reviewed the journal’s formatting templates and have made all necessary adjustments to ensure that the manuscript fully complies with the prescribed style, including file naming conventions.

**Comment 2：Please note that PLOS One has specific guidelines on code sharing for submissions in which author-generated code underpins the findings in the manuscript. In these cases, we expect all author-generated code to be made available without restrictions upon publication of the work.**

**Response 2:** Thank you for highlighting PLOS One’s policy on code sharing. We fully support the journal’s commitment to transparency and reproducibility. In this study, statistical analyses were performed using SPSS, which operated primarily through a graphical interface rather than script-based coding, we have illustrated our operation process through screenshots. For modeling and evaluation steps—including ROC curves, calibration curves, nomogram construction, and clinical benefit curves—we used STATA and would provide the exact scripts used for these procedures. Additionally, variable selection via LASSO regression and Kaplan-Meier survival analyses were conducted using R, and we will also share fully executable R code supporting these elements. All code will be made openly available upon publication in a manner consistent with best practices to facilitate reuse and reproducibility.

**Comment 3：Please state what role the funders took in the study. If the funders had no role, please state: "The funders had no role in study design, data collection and analysis, decision to publish, or preparation of the manuscript."**

**If this statement is not correct you must amend it as needed. Please include this amended Role of Funder statement in your cover letter; we will change the online submission form on your behalf.**

**Response 3:** We confirm that the funders had no role in the study design, data collection and analysis, decision to publish, or preparation of the manuscript. The amended Role of Funder statement, as required, was provided below for inclusion in the cover letter: "The funders had no role in study design, data collection and analysis, decision to publish, or preparation of the manuscript."

**Comment 4：Please note that funding information should not appear in any section or other areas of your manuscript. We will only publish funding information present in the Funding Statement section of the online submission form. Please remove any funding-related text from the manuscript.**

**Response 4:** We sincerely appreciate your guidance regarding the proper placement of funding information. In accordance with PLOS One’s policy, we have thoroughly reviewed the manuscript and removed all mentions of funding support, including the acknowledgment of Dr. Wen’s grant from the National Natural Science Foundation of China (Grant No. 82474299) and the description of his research role, which previously appeared in the Authors’ Contributions section. This information will be provided exclusively in the Funding Statement section of the submission system. In this edition, manuscript did not contain any funding-related text.

**Comment 5：Please include a separate caption for each figure in your manuscript.**

**Response 5:** Thank you for your reminder. We have carefully reviewed the journal’s figure guidelines and have now included a separate, descriptive caption for each figure in the manuscript. All captions have been placed at the end of the main text, as required, and are also clearly indicated on pages 40 and 41 for ease of reference during review. The captions provide full and clear descriptions of each figure’s content.

**Comment 6：Please remove your figures from within your manuscript file, leaving only the individual TIFF/EPS image files, uploaded separately. These will be automatically included in the reviewers’ PDF.**

**Response 6:** We thank you for your clear instruction regarding figure preparation and submission. We have now removed all figures from within the manuscript file. Each figure has been saved and uploaded separately as an individual TIFF/EPS file, as required. The manuscript text now refers to each figure by its label, and all figure files have been prepared in accordance with PLOS One’s guidelines to ensure proper inclusion in the reviewers’ PDF.

**Comment 7：Please remove all personal information, ensure that the data shared are in accordance with participant consent, and re-upload a fully anonymized data set.**

**Response 7:** We thank you for your important reminder regarding data anonymization and participant confidentiality. We confirm that the data used in this study were obtained from the MIMIC-IV database, which is a publicly available, fully de-identified critical care database. All protected health information has been removed in accordance with ethical and legal requirements prior to public release of the database. We have conducted an additional thorough review of our dataset and can confirm that it contains no personally identifiable information. The use of the MIMIC-IV database was approved by the institutional review boards of Beth Israel Deaconess Medical Center and the Massachusetts Institute of Technology, and the requirement for individual patient consent was waived due to the retrospective and de-identified nature of the data. We have re-uploaded the fully anonymized dataset to ensure full compliance with PLOS One’s data sharing policy.

**Comment 8：Please include captions for your Supporting Information files at the end of your manuscript, and update any in-text citations to match accordingly. Please see our Supporting Information guidelines for more information.**

**Response 8:** Thank you for this important reminder. We have now included detailed captions for all Supporting Information files at the end of the manuscript, as specified on pages from 42 to 43. All relevant in-text citations have also been updated to correspond accurately to these supporting materials. We have carefully followed PLOS One’s Supporting Information guidelines to ensure full compliance.

**Comment 9：If the reviewer comments include a recommendation to cite specific previously published works, please review and evaluate these publications to determine whether they are relevant and should be cited. There is no requirement to cite these works unless the editor has indicated otherwise.**

**Response 9:** Thank you for your clear guidance on this matter. We have carefully reviewed all comments provided by the reviewers and confirm that no specific recommendations were made regarding the citation of additional previously published works. We have therefore not added any further citations in response to reviewer suggestions.

**Responses to Reviewer #1:**

**Comment 1a1：Authors decided to mention but questionably apply to the following text three subclasses of decompensated advanced chronic liver disease (dACLD): stable dACLD, unstable dACLD and ACLF. This division majorly overlaps with the PREDICT study methodology. If considered relevant for the text, amend accordingly. If not, consider erasing (otherwise, it should to be absolutely clear how many patients were from which of the subgroups plus ACLF. If authors decided otherwise, it is acceptable but should be pathophysiologically explained and challenged against existing tools).**

**Response 1a1:** We sincerely thank the reviewer for their insightful and constructive comment regarding the subclassification of decompensated advanced chronic liver disease (dACLD). We fully agree that the distinction between stable dACLD, unstable dACLD, and ACLF aligns closely with the PREDICT study methodology. In response to this valuable feedback, we have revised the manuscript to focus specifically on ACLF, as the high short-term mortality in end-stage cirrhosis is predominantly associated with ACLF—a point strongly supported by the EASL Clinical Practice Guidelines (European Association for the Study of the Liver, J Hepatol. 2023;79(2):461–491), which state that mortality in cirrhosis is largely attributable to acute-on-chronic liver failure. We have therefore removed references to stable and unstable dACLD to improve conceptual clarity and thematic consistency. The corresponding changes have been made in the Introduction (Page 4, Lines 5–7).

**Comment 1a2：I suggest in Intro to direct readers' attention by describing those aspects of dACLD from the literature which are relevant to the study: Items from the Intro should align with items in the Table 1 and subsequent Tables with results (mismatches: SGA, hepatic encephalopathy, HRS, frailty, serial liver stiffness measurements, etc.). It is futile to devote equal space to the variables authors know they are not going to include in their analysis and those included; moreover, as regards the latter, readers should gradually understand why authors included recorded variables both biologically, and based on the existing literature.**

**Response 1a2:** We are deeply grateful to the reviewer for this highly constructive suggestion, which has significantly improved the focus and clarity of our Introduction. We fully agree that the introductory narrative should align closely with the variables ultimately analyzed and presented in our results, such as those summarized in Table 1. Accordingly, we have removed extended discussions of scoring systems and clinical markers not directly relevant to our model—including SGA, hepatic encephalopathy, HRS, frailty, and serial liver stiffness measurements—to avoid diverting the reader’s attention, with these revisions concentrated on pages 5–7 of the manuscript.

Furthermore, we have refocused the Introduction to provide a clearer biological and clinical rationale for the variables incorporated in our analysis. We now specifically introduce and explain established ACLF-specific prognostic tools such as the CLIF-OF and CLIF-C ACLF scores, which serve as meaningful benchmarks for comparing our model's performance [1, 2]. Additionally, we emphasize the pathophysiological and empirical justification for each selected predictor—encompassing both individual and composite indicators—based on prior experimental and clinical research in ACLF. This approach ensures that readers can gradually comprehend the scientific and clinical reasoning behind our variable selection, thereby creating a much stronger logical connection between the background, our methodological choices, and the results.

References

[1]. Arroyo V, Jalan R. Acute-on-chronic liver failure: definition, diagnosis, and clinical characteristics. Semin Liver Dis. 2016;36(2):109-16.

[2]. Barosa R, Roque Ramos L, Patita M, Nunes G, Fonseca J. CLIF-C ACLF score is a better mortality predictor than MELD, MELD-Na and CTP in patients with Acute on chronic liver failure admitted to the ward. Rev Esp Enferm Dig. 2017;109(6):399-405.

**Comment 2：It should be mentioned why authors have not included organ/system dysfunction beyond cardiac (described as cardiovascular) and renal: what about those defining ACLF? Also, one would expect including portal hypertensive bleeding in the introductory outline.**

**Response 2:** We sincerely thank the reviewer for raising these critical points regarding the scope of organ dysfunction and the inclusion of portal hypertensive bleeding. We appreciate the opportunity to clarify these important aspects of our study.

Regarding the inclusion of other organ dysfunctions beyond cardiac and renal systems, we did initially consider and extract available data on additional complications (e.g., respiratory, neurological, and coagulation disorders) as part of our comprehensive statistical analysis. However, as summarized in Tables 1 (see below), the prevalence of these additional organ failures was relatively low in our cohort, and crucially, they did not demonstrate statistically significant differences between the short-term mortality and non-mortality groups. Therefore, although clinically relevant in broader definitions of ACLF, these variables were not included as predictors in our final model. We acknowledge this limitation and have added a sentence in the Discussion to note that future models might be strengthened by the inclusion of such parameters in larger, more granular datasets.

Concerning portal hypertensive bleeding, we fully agree with the reviewer that it represents a key factor influencing short-term mortality in end-stage cirrhosis and ACLF. Unfortunately, after thorough data extraction and coding from the MIMIC-IV database, we were unable to identify reliably documented variables specifically capturing portal hypertensive bleeding events. This represents a recognized limitation of utilizing large public critical care databases, which often lack detailed hepatology-specific phenotyping. Nonetheless, we agree with the reviewer that portal hypertension plays a central role in the pathophysiology of ACLF. Accordingly, we have expanded the Discussion section to emphasize the systemic role of portal hypertension and how several of the included variables (such as platelet count and INR) may indirectly reflect its contribution, even if direct measures were unavailable.

**Table1. Baseline Analysis of the training cohort**

| Variable | Total Patients (n=740) | Survival (n=536) | Dead (n=204) | P value |
| --- | --- | --- | --- | --- |
| **Co-morbidity** |  |  |  |  |
| Myocardial infarct |  |  |  |  |
| Yes | 42 (5.7%) | 26 (4.9%) | 16 (7.8%) | 0.116 |
| No | 698 (94.3%) | 510 (95.1%) | 188 (92.2%) |  |
| Congestive heart failure |  |  |  |  |
| Yes | 146 (19.7%) | 102 (19%) | 44 (21.6%) | 0.438 |
| No | 594 (80.3%) | 434 (81%) | 160 (78.4%) |  |
| Peripheral vascular disease |  |  |  |  |
| Yes | 50 (6.8%) | 37 (6.9%) | 13 (6.4%) | 0.797 |
| No | 690 (93.2%) | 499 (93.1%) | 191 (93.6%) |  |
| Cerebrovascular disease |  |  |  |  |
| Yes | 54 (7.3%) | 32 (6%) | 22 (10.8%) | 0.024 |
| No | 686 (92.7%) | 504 (94%) | 182 (89.2%) |  |
| Chronic pulmonary disease |  |  |  |  |
| Yes | 181 (24.5%) | 137 (25.6%) | 44 (21.6%) | 0.259 |
| No | 559 (75.5%) | 399 (74.4%) | 160 (78.4%) |  |
| Peptic ulcer disease |  |  |  |  |
| Yes | 56 (7.6%) | 48 (9%) | 8 (3.9%) | 0.021 |
| No | 684 (92.4%) | 488 (91%) | 196 (96.1%) |  |
| Renal disease |  |  |  |  |
| Yes | 172 (23.2%) | 109 (20.3%) | 63 (30.9%) | 0.002 |
| No | 568 (76.8%) | 427 (79.7%) | 141 (69.1%) |  |
| Severe liver disease |  |  |  |  |
| Yes | 430 (58.1%) | 304 (56.7%) | 126 (61.8%) | 0.214 |
| No | 310 (41.9%) | 232 (43.3%) | 78 (38.2%) |  |

^a^*P* value: probability value; ^b^RBC: red blood cell; ^c^ALT: alanine aminotransferase; ^d^ALP: alkaline Phosphatase; ^e^AST: aspartate aminotransferase; ^f^INR: international normalized ratio; ^g^WBC: white blood cell; ^h^BUN: blood urea nitrogen.

**Comment 3：Power analysis?**

**Response 3:** Thanks to this crucial methodological point. As this is a retrospective study utilizing the fixed MIMIC-IV database, a formal a priori power analysis conducted prior to data collection was not feasible. The study included all eligible patients from the database who met our inclusion and exclusion criteria, resulting in a final cohort of 740 subjects.

However, to directly address the reviewer's concern regarding statistical power, we have now performed a post-hoc power analysis using DeLong's test for the area under the ROC curve (AUC) in R (using the pROC package). Based on our observed AUC of 0.846, a significance level (α) of 0.05, and with 204 events (deaths) and 536 controls (survivors), the analysis revealed that our study possesses >99.9% statistical power to detect that the model's performance is significantly better than chance (AUC > 0.5).This result confirms that our sample size is not only adequate but indeed provides exceedingly high power to robustly support the primary findings of this study. We have added a brief statement regarding this post-hoc power assessment in the “Statistical Analysis” section of the revised manuscript (Page 11, Line 3 - 6).

**Comment 4：Repeated hospitalisations before index one: The number of previous hospital admissions with dACLD is essential variable to be included in the final analysis because inherent prognosis differs substantially.**

**Response 4:** We sincerely thank the reviewer for highlighting the potential importance of prior hospitalizations as a prognostic variable. In our analysis, we did indeed collect and examine the number of previous hospital admissions for dACLD. The statistical results indicated that a higher number of prior hospitalizations was significantly associated with a reduced risk of short-term mortality (OR = 0.681, 95% CI: 0.592–0.784, *p* < 0.001).

However, after careful consideration, we decided not to include this variable in the final model. While statistically significant, this counterintuitive protective effect may reflect unmeasured confounding factors, such as selection bias (e.g., patients with repeated admissions might have better access to care or greater resilience to acute deteriorations) or differences in underlying disease trajectories. Moreover, since our objective was to develop a parsimonious and physiologically interpretable model focused on objective clinical indicators at ICU admission, we prioritized variables with clearer biological plausibility and wider consensus in prognostic models for ACLF.

**Comment 5：Again - bilirubin is not a typical marker of synthesis and this repeated claim might shed a doubt on the whole work; akin to the claim on INR and coagulation vs synthesis. Do authors know if patients were on anticoagulants at the time of recording INR?**

**Response 5:** We sincerely thank the reviewer for raising these critical methodological points, which have significantly strengthened the accuracy and interpretation of our work. We fully agree that bilirubin should not be characterized primarily as a marker of hepatic synthesis. In direct response to this comment, we have carefully revised the relevant descriptions throughout the manuscript to more accurately reflect that bilirubin is a marker of hepatic excretion and cholestatic function rather than synthetic capacity. These corrections have been made in the “Data Collection and Processing” section (Lines 5–9).

Regarding the potential influence of anticoagulant therapy on INR values, we thank the reviewer for highlighting this important confounding factor. We have now extracted additional data on anticoagulant use at the time of ICU admission. Subsequent subgroup analyses were performed comparing patients with and without anticoagulation. The results consistently demonstrated that INR remained a strong and independent predictor of short-term mortality in both subgroups, and its prognostic value was not materially altered by anticoagulant use. Risk stratification based on INR remained consistent across anticoagulation subgroups, supporting the robustness of this variable in our model (Fig 1).

Fig 1. Kaplan-Meier Analysis by Anticoagulant Use. (A) Anticoagulant Group: Short-term survival in severe cirrhosis stratified by international normalized ratio (INR) of prothrombin. (B) Non-Anticoagulant Group: Short-term survival in severe cirrhosis stratified by international normalized ratio (INR) of prothrombin.

**Comment 6：Why have authors not included (along with or instead of APS, OASIS, MELD) ACLF/CLIF OF as the sole most logical and the most important comparator?**

**Response 6:** We sincerely thank the reviewer for this critical and insightful comment. We fully agree that comparison with ACLF-specific prognostic scores is essential for validating the clinical relevance of our model. In direct response to this suggestion, we have now included the CLIF-OF and CLIF-C ACLF scores as key comparators in our revised analysis.

As shown in page 22 and discrimination performance [When benchmarked against conventional scoring systems, our model exhibited superior discriminative ability: derivation cohort AUC improved by 0.015–0.109 compared to MELD (AUC 0.737) ，MELD-Na (AUC 0.741)，CLIF-OF(AUC 0.817) and CLIF-C ACLF (AUC 0.831) while validation cohort AUC exceeded both scores by 0.059–0.029 (MELD: 0.784; MELD-Na: 0.792; CLIF-OF: 0.813; CLIF-C ACLF: 0.814 ) ] of our revised manuscript, the performance of our proposed model was favourably compared to these established ACLF-specific scoring systems. Notably, our model demonstrated superior discriminative ability for predicting short-term mortality in this critically ill population, reinforcing its potential clinical utility(Fig 2).

Fig 2. Kaplan-Meier Analysis by Anticoagulant Use. (A) Anticoagulant Group: Short-term survival in severe cirrhosis stratified by international normalized ratio (INR) of prothrombin. (B) Non-Anticoagulant Group: Short-term survival in severe cirrhosis stratified by international normalized ratio (INR) of prothrombin.

**Comment 7：Etiology of ACLD was not mentioned: e.g., how many patients had severe alcohol-associated hepatitis as the trigger of acute decompensation? The prognosis of these patients may be different from that in MASH-cirrhosis, autoimmune-cirrhosis, etc.**

**Response 7:** We sincerely thank the reviewer for raising this important point regarding the potential influence of cirrhosis etiology on prognostic accuracy. In direct response to this comment, we conducted a detailed subgroup analysis comparing outcomes between patients with alcohol-related cirrhosis and those with non-alcohol-related causes. Among the 545 patients with alcoholic cirrhosis, 161 (29.5%) experienced short-term mortality, compared to 145 of 499 (29.1%) in the non-alcoholic group, with no statistically significant difference in mortality risk (P = 0.918). Furthermore, evaluation of model performance within each subgroup demonstrated that our predictive model consistently achieved superior discrimination and provided greater clinical net benefit on decision curve analysis compared to existing scores, regardless of underlying etiology. These findings have been added as a new subsection ("Performance Across Etiological Subtypes") in the Results and are further supported by additional figures (Fig 3-4).

Fig 3. Relationship between alcoholic liver disease, non-alcoholic liver disease and short-term mortality. In the subgroup analysis, there were 545 patients in the alcoholic cirrhosis group, among whom 161 (29.5%) experienced short-term mortality and 384 (70.5%) survived in the short term. In the non-alcoholic cirrhosis group, there were 499 patients, with 145 (29.1%) experiencing short-term mortality and 354 (70.9%) surviving in the short term. Statistical analysis showed no significant difference in short-term mortality between the two groups (P = 0.918).

Fig 4. Comparative Evaluation of Predictive Models and Clinical Benefit Analysis for Short-Term Mortality in Patients with Severe Alcoholic vs. Non-Alcoholic Cirrhosis.(A) Comparison of AUC values among multiple models in the alcoholic cirrhosis group.(B) Comparison of AUC values among multiple models in the non-alcoholic cirrhosis group.(C) Clinical decision curve analysis for multiple models in the alcoholic cirrhosis group.(D) Clinical decision curve analysis for multiple models in the non-alcoholic cirrhosis group.

**Comment 8：Including malignancies. This is the most problematic point. Prognostic systems in ACLF (which is implicitly the cohort in question - by the words of authors: ICU-hospitalised patients with decompensated cirrhosis). As ACLF prognostic scores DO NOT include malignancies and ACLF should be the benchmark syndrome against which most of the readers will intuitively or explicitly measure the results, It should be addressed in the necessary detail. 1. If authors want to add to existing tools, they should exclude malignancies. 2. If they want to build a new prognostic system for patients with both - acutely decompensated cirrhosis and malignancy, they should recalculate the results in this subgroup for which, in my opinion, the numbers are small.**

**Response 8:** We sincerely thank the reviewer for raising this critical point regarding the inclusion of patients with malignancies. We fully agree that the prognosis of these patients is considerably more complex, as outcomes are often influenced more by oncological progression than by liver-specific pathophysiological processes. This perspective is well-noted and highly valuable.

We have excluded all 29 patients with metastatic malignancies from our cohort. To maintain statistical power and ensure a robust sample size, we supplemented the dataset by identifying additional patients with decompensated cirrhosis without malignancies from the same database who met the original inclusion criteria. The prognostic model was subsequently rebuilt using this refined cohort, which now more accurately represents a population relevant to liver-specific mortality risk. This adjustment not only aligns with standard practices in ACLF prognostic research but also strengthens the clinical applicability of our model to typical cirrhotic patients without confounding oncological conditions.

**Comment 9：Authors discarded statistically significant INR difference (Table 1) as not important only in passing: why they think so?It is important to substantiate this very important point factually, not only by sheer "We think". INR translates to all the results which follow including 3.2Model development and 3.5 INR Stratification analysis. The same with Renal disease.**

**Response 9:** We sincerely thank the reviewer for emphasizing the importance of substantiating our treatment of variables showing baseline imbalance. We appreciate the opportunity to clarify our rationale regarding the observed differences in INR and renal disease between the training and validation cohorts.

As noted in the revised manuscript (Page 11, Lines 14–21), although INR and renal disease showed statistically significant differences in distribution between the two cohorts (p < 0.05), we wish to emphasize that these variables were not “discarded” or ignored. Rather, we conducted several additional analyses to evaluate their potential impact. First, the overall randomized allocation of patients supports that this imbalance was likely due to chance rather than systematic bias. More importantly, we performed rigorous validation steps—including calibration curves, ROC analysis, and specific adjustment for anticoagulant use in the case of INR—which collectively demonstrated that the discriminative and predictive performance of the model remained robust in both cohorts. These results indicated that the observed baseline differences did not materially affect the validity or generalizability of the model.

**Comment 10: Discussion is too brief. Authors should expose their deep knowledge point by point of their results.**

**Response 10:** We sincerely thank the reviewer for pointing out the need for a more in-depth discussion of our results. We agree that the Discussion section should provide a thorough and point-by-point interpretation of the key findings, linking them back to the existing literature and clinical implications. In response to this suggestion, we have substantially expanded the Discussion section to better reflect our understanding of the results. The modifications are as follows:

**Key Findings and Model Development**

This study presents the first comprehensive predictive model for short-term mortality risk in patients with severe cirrhosis, incorporating an analysis of short-term prognosis and evaluating the prognostic significance of the international normalized ratio (INR). Our findings demonstrate that the prediction model exhibits excellent sensitivity and specificity, with short-term prognosis being significantly associated with multiple factors including age, AST/ALT, INR, creatinine, platelet count, white blood cell count, total bilirubin levels, white blood cell count, and the presence of comorbidities such as peptic ulcer disease . Elevated age, INR, creatinine, white blood cell count, total bilirubin, and AST/ALT ratio were correlated with poorer prognosis and increased short-term mortality risk, whereas decreased platelet levels were also associated with worse outcomes. Interestingly, the presence of peptic ulcer disease was linked to a reduced risk of mortality compared to its absence.Furthermore, significant differences in survival outcomes were observed across different INR ranges, with risk stratification effectively delineated into three distinct intervals. Within these strata, a higher INR was consistently associated with increased short-term mortality risk—regardless of anticoagulant therapy use.

**Multidimensional Predictors of Short-Term Mortality in Severe Cirrhosis**

The high short-term mortality risk and poor survival prognosis in severe cirrhosis patients underscore the clinical importance of accurate risk prediction. Our analysis revealed that each additional year of age increased short-term mortality risk by 1.04-fold, consistent with existing literature documenting the critical impact of age-related physiological changes on cirrhosis outcomes. These changes include deterioration in circulatory dynamics, organ function, and immune competence, all of which significantly influence the development of cirrhosis-related complications [1]. The strong association between age and prognosis in our study confirms its value as a reliable predictor of short-term mortality in severe cirrhosis.

Renal function emerged as another crucial prognostic factor, with elevated creatinine levels demonstrating significant predictive value. While the precise mechanisms linking cirrhosis to renal impairment remain incompletely understood, our findings support existing research identifying multiple contributing factors: hypovolemia (50% of cases), intrinsic structural kidney damage (30%), hepatorenal syndrome (15-20%), and postrenal obstruction (<1%) [2,3]. These diverse pathways collectively impair renal excretory function, leading to creatinine elevation and corresponding increases in mortality risk.

Hematological parameters provided additional prognostic insights. Thrombocytopenia showed an inverse relationship with survival, potentially attributable to both decreased platelet production (mediated through reduced thrombopoietin activity in fibrotic livers [4]) and increased platelet destruction (via hypersplenism and immune-mediated clearance [5,6]). White blood cell count served as an independent predictor of mortality, reflecting the high prevalence (24-29%) of bacterial infections in advanced cirrhosis and their substantial contribution to poor outcomes [7,8]. The inclusion of this parameter addresses a notable gap in previous prediction models that overlooked infection-related mortality.

Total bilirubin levels demonstrated significant prognostic value, as a key indicator of hepatic metabolic function, total bilirubin level reflects abnormalities in liver function, with both direct and indirect bilirubin often exhibiting elevated levels in patients with cirrhosis. The underlying mechanisms primarily involve the following aspects: First, portal hypertension-induced hypersplenism promotes excessive destruction of red blood cells in the spleen, leading to chronic hemolysis. Splenic congestion and enlargement accelerate erythrocyte breakdown [9]. In addition, portal hypertension alters hemorheological parameters—including erythrocyte membrane lipid composition and protein conformation—which reduce red blood cell deformability and increase their susceptibility to destruction within the microcirculation [10]. Furthermore, structural and functional abnormalities in the microvasculature under portal hypertension contribute to impaired blood flow, exposing red blood cells to mechanical stress and further promoting hemolysis. As bilirubin is a metabolic product of hemoglobin breakdown, hemolysis significantly increases its circulating levels. On the other hand, intrahepatic cholestasis and altered portal blood flow in cirrhosis impair hepatic clearance of bilirubin, leading to elevated direct bilirubin levels [11]. Additionally, during the decompensated stage, impaired glucuronidation of indirect bilirubin reduces its conjugation capacity, also contributing to increased indirect bilirubin levels [12,13]. The resultant rise in both direct and indirect bilirubin elevates total bilirubin levels, which our study confirms as a significant predictor of short-term mortality in severe cirrhosis.

In our predictive model, we observed that critically ill cirrhotic patients with peptic ulcer disease exhibited a lower short-term mortality compared to those without peptic ulcers. This finding appears to contradict certain studies indicating an increased risk of death within 90 days after hospitalization for peptic ulcer in cirrhotic patients [14]. The discrepancy may be attributed to several factors. Firstly, the subgroup of patients with peptic ulcers might have had less severe overall disease. Compared to complications such as esophagogastric variceal bleeding, peptic ulcer hemorrhage is associated with a lower risk of mortality, which could largely explain our results. Secondly, cirrhotic patients diagnosed with peptic ulcers likely received comprehensive therapeutic regimens, including non-steroidal anti-inflammatory drugs, mucosal protectants, proton pump inhibitors, acid suppressants, antibiotics, prophylactic low-dose aspirin, antifibrinolytic agents, as well as hemostatic and transfusion therapies for bleeding ulcers. These interventions may have reduced the risk of hemorrhage and other complications in severe cirrhosis, thereby potentially contributing to a lower short-term mortality in this patient group [15,16].

The International Normalized Ratio (INR) is an established predictor of bleeding in patients with liver and acute diseases[17]. Our analysis confirms that INR levels also effectively predict short-term mortality in critically ill cirrhotic patients, with significant disparities across INR strata. This association stems from the liver’s central role in synthesizing coagulation proteins—such as albumin, proteins C and S, and clotting factors—whose production is impaired in ACLF, leading to elevated INR [18,19]. Concurrent organ failures, such as acute kidney injury, may exacerbate coagulation dysfunction through metabolic disturbances and impaired toxin clearance. Although studies like that of Amber Afzal et al. suggest that INR-associated bleeding risk varies with etiology and medication (e.g., warfarin use leading to exponential risk increase above INR 3)[20], our subgroup analyses demonstrated that INR stratification consistently discriminated short-term mortality risk regardless of anticoagulant use. Higher INR levels correlated with increased bleeding risk and worse prognosis, consistent with existing literature.

**References**

[1].	Kamimura K, Sakamaki A, Kamimura H, Setsu T, Yokoo T, Takamura M, Terai S. Considerations of elderly factors to manage the complication of liver cirrhosis in elderly patients. World J Gastroenterol. 2019;25(15):1817-27.

[2].	Belmonte R, Silva-Rodriguez M, Barbé F, Bensenane M, Haghenejad V, Vrillon I, et al. Multiparametric renal function assessment in cirrhotic patients shows high prevalence of medically actionable changes in multiple modules. Hepatol Res. 2024;54(11):1035-48.

[3].	Ozturk NB, Dinc EJ, Swami A, Gurakar A. Acute Kidney Injury and Hepatorenal Syndrome in Patients with Cirrhosis. J Clin Med. 2023;13(1).

[4].	Kwaan H, Lisman T, Medcalf RL. Fibrinolysis: Biochemistry, Clinical Aspects, and Therapeutic Potential. Semin Thromb Hemost. 2017;43(2):113-4.

[5].	Chen SH, Tsai SC, Lu HC. Platelets as a Gauge of Liver Disease Kinetics? Int J Mol Sci. 2022;23(19).

[6].	Baccini V, Geneviève F, Jacqmin H, Chatelain B, Girard S, Wuilleme S, et al. Platelet Counting: Ugly Traps and Good Advice. Proposals from the French-Speaking Cellular Hematology Group (GFHC). J Clin Med. 2020;9(3).

[7].	Albillos A, Lario M, Álvarez-Mon M. Cirrhosis-associated immune dysfunction: distinctive features and clinical relevance. J Hepatol. 2014;61(6):1385-96.

[8].	Piano S, Bunchorntavakul C, Marciano S, Rajender Reddy K. Infections in cirrhosis. Lancet Gastroenterol Hepatol. 2024;9(8):745-57.

[9].	Gerlag PG, Lobatto S, Driessen WM, Deckers PF, Van Hooff JP, Schröder E, et al. Hepatic sinusoidal dilatation with portal hypertension during azathioprine treatment after kidney transplantation. J Hepatol. 1985;1(4):339-48.

[10].	Sanyal AJ, Freedman AM, Purdum PP, Shiffman ML, Luketic VA. The hematologic consequences of transjugular intrahepatic portosystemic shunts. Hepatology. 1996;23(1):32-9.

[11].	Ohkubo A. [Bilirubin metabolism in liver cirrhosis]. Nihon Rinsho. 1994;52(1):138-44.

[12].	Aravinthan AD, Alexander GJ. Hepatocyte senescence explains conjugated bilirubinaemia in chronic liver failure. J Hepatol. 2015;63(2):532-3.

[13].	Lee HA, Jung JY, Lee YS, Jung YK, Kim JH, An H, et al. Direct Bilirubin Is More Valuable than Total Bilirubin for Predicting Prognosis in Patients with Liver Cirrhosis. Gut Liver. 2021;15(4):599-605.

[14].	Holland-Bill L, Christiansen CF, Gammelager H, Mortensen RN, Pedersen L, Sørensen HT. Chronic liver disease and 90-day mortality in 21,359 patients following peptic ulcer bleeding--a Nationwide Cohort Study. Aliment Pharmacol Ther. 2015;41(6):564-72.

[15].	Kamada T, Satoh K, Itoh T, Ito M, Iwamoto J, Okimoto T, et al. Evidence-based clinical practice guidelines for peptic ulcer disease 2020. J Gastroenterol. 2021;56(4):303-22.

[16].	Vakil N. Peptic Ulcer Disease: A Review. Jama. 2024;332(21):1832-42.

[17].	Raza S, Pinkerton P, Hirsh J, Callum J, Selby R. The historical origins of modern international normalized ratio targets. J Thromb Haemost. 2024;22(8):2184-94.

[18].	Northup PG, Sundaram V, Fallon MB, Reddy KR, Balogun RA, Sanyal AJ, et al. Hypercoagulation and thrombophilia in liver disease. J Thromb Haemost. 2008;6(1):2-9.

[19].	Tripodi A, Mannucci PM. The coagulopathy of chronic liver disease. N Engl J Med. 2011;365(2):147-56.

[20].	Afzal A, Gage BF, Suhong L, Schoen MW, Korenblat K, Sanfilippo KM. Different risks of hemorrhage in patients with elevated international normalized ratio from chronic liver disease versus warfarin therapy, a population-based retrospective cohort study. J Thromb Haemost. 2022;20(7):1610-7.

**Comment 11： Apart from above mentioned points (ACLF!!!), they should discuss portal hypertension as the point which is probably not captured enough in their system.**

**Response 11:** We are very grateful to the reviewer for emphasizing the critical role of portal hypertension in the pathophysiology and outcomes of ACLF. We agree that it is a key factor that merits detailed discussion. In direct response to this comment, we have now added a dedicated paragraph in the Discussion section (Page 27-28), that explicitly addresses the central role of portal hypertension. The modifications are as follows:

**Portal hypertension plays a central role**

Although portal hypertension (PHT) was not directly incorporated into our model due to limitations in the database, it nonetheless demonstrated a pivotal role in the progression to acute and chronic liver failure in cirrhosis, with its central importance in ACLF being particularly evident. PHT may serve as an underlying pathway connecting multiple observations within our study; it is not merely an isolated hemodynamic phenomenon, but rather a systemic disorder whose effects are reflected across a range of biomarkers we analyzed. A key finding was that thrombocytopenia—significantly associated with short-term mortality in our cohort—is highly prevalent in cirrhotic patients (reaching 77.9%), and is closely linked to PHT-induced hypersplenism, which promotes platelet sequestration and destruction [1,2]. Similarly, accelerates red blood cell breakdown. This PHT-related hemolytic process provides a plausible mechanism for the elevated bilirubin observed in our ACLF patients, which has been histopathologically correlated with impaired bile processing and poor prognosis. Furthermore, hemolysis adds to the bilirubin burden, acting as an “accelerator” of hyperbilirubinemia in ACLF [3]. Beyond hematologic effects, PHT compromises intestinal barrier integrity, facilitating bacterial translocation, endotoxemia, and systemic inflammation [4], thereby accounting for the variations in white blood cell counts noted in our analysis. PHT also exacerbates direct liver injury: recent evidence indicates that it promotes hepatic translocation of pathogens such as H. *pylori*, which can induce NLRP3-mediated pyroptosis and elevate aminotransferase levels [5]. Consistently, a study on TIPS showed that reducing portal pressure led to marked improvements in ALT, AST, and INR [6], reinforcing that higher PHT exacerbates hepatocellular damage and dysfunction. Additionally, PHT drives severe extrahepatic complications such as hepatorenal syndrome (suggested by rising creatinine) and peptic ulcer disease through mechanisms like gastric mucosal congestion. In summary, although not directly quantified, the “ghost” of PHT permeates nearly all the biomarkers we studied; it likely interconnects hypersplenism, hepatic decompensation, systemic inflammation, and end-organ damage, serving as a core pathophysiological mechanism in ACLF that is further corroborated by our findings.

**References**

[1].	Fierro-Angulo OM, González-Regueiro JA, Pereira-García A, Ruiz-Margáin A, Solis-Huerta F, Macías-Rodríguez RU. Hematological abnormalities in liver cirrhosis. World J Hepatol. 2024;16(9):1229-44.

[2].	Yoshida H, Shimizu T, Yoshioka M, Matsushita A, Kawano Y, Ueda J, et al. The Role of the Spleen in Portal Hypertension. J Nippon Med Sch. 2023;90(1):20-5.

[3].	Nacif LS, Aquino F, Tanigawa RY, Zanini LY, Pinheiro RS, Rocha-Santos V, et al. Histopathologic Evaluation of Acute on Chronic Liver Failure. Transplant Proc. 2020;52(5):1325-8.

[4].	El-Reshaid K, Alabdulghani F, Al-Bader S. Partial Splenic Embolization for Hypersplenism Associated with Steatohepatitis in a Hemodialysis Patient. Saudi J Kidney Dis Transpl. 2021;32(1):255-60.

[5].	Trebicka J, Bork P, Krag A, Arumugam M. Utilizing the gut microbiome in decompensated cirrhosis and acute-on-chronic liver failure. Nat Rev Gastroenterol Hepatol. 2021;18(3):167-80.

[6].	Hu J, Gao L, Maswikiti EP, Li X, Gu B, Yu Y, et al. Portal hypertension aggravates Helicobacter pylori induced liver injury in mice via activating pyroptosis pathway. Sci Rep. 2025;15(1):11820

**Comment 12：Please, discuss in even more detail the contribution of haemolysis to the hyperbilirubinaemia with the references.**

**Response 12:** We sincerely thank the reviewer for this insightful suggestion. We fully agree that a more detailed discussion on the potential contribution of hemolysis to hyperbilirubinaemia is essential for a comprehensive pathophysiological interpretation of our findings. We have incorporated an in-depth discussion as suggested, specifically in lines 13–14 on page 29 to line 9 on page 30 of the revised manuscript.

Total bilirubin levels demonstrated significant prognostic value, as a key indicator of hepatic metabolic function, total bilirubin level reflects abnormalities in liver function, with both direct and indirect bilirubin often exhibiting elevated levels in patients with cirrhosis. The underlying mechanisms primarily involve the following aspects: First, portal hypertension-induced hypersplenism promotes excessive destruction of red blood cells in the spleen, leading to chronic hemolysis. Splenic congestion and enlargement accelerate erythrocyte breakdown. In addition, portal hypertension alters hemorheological parameters—including erythrocyte membrane lipid composition and protein conformation—which reduce red blood cell deformability and increase their susceptibility to destruction within the microcirculation. Furthermore, structural and functional abnormalities in the microvasculature under portal hypertension contribute to impaired blood flow, exposing red blood cells to mechanical stress and further promoting hemolysis. As bilirubin is a metabolic product of hemoglobin breakdown, hemolysis significantly increases its circulating levels.On the other hand, intrahepatic cholestasis and altered portal blood flow in cirrhosis impair hepatic clearance of bilirubin, leading to elevated direct bilirubin levels. Additionally, during the decompensated stage, impaired glucuronidation of indirect bilirubin reduces its conjugation capacity, also contributing to increased indirect bilirubin levels. The resultant rise in both direct and indirect bilirubin elevates total bilirubin levels, which our study confirms as a significant predictor of short-term mortality in severe cirrhosis.

**Comment 13：I do not agree that PPI improve prognosis. Overall, discussion of this most controversial (and maybe most important) finding of the study deserves in-depth discussion disposing your detailed knowledge**

**Response 13:** We sincerely thank the reviewer for raising this crucial point. We fully agree that the observed association between peptic ulcer disease and improved prognosis is both controversial and clinically significant, and that it warrants a thorough and nuanced discussion rather than a simplistic causal interpretation.

In direct response to your comment, we have substantially expanded our discussion of this finding in the revised manuscript (Page 30). We explicitly state that we do not assert a direct causal or protective effect of peptic ulcers themselves. Instead, we propose that the observed association is likely explained by confounding factors. Specifically, we elaborate that: 1) patients with diagnosed peptic ulcers may represent a subgroup with a different overall disease profile and access to structured care; and 2) these patients invariably receive multi-faceted medical management—including acid-suppressive therapy, H. *pylori* eradication, mucosal protectants, and close monitoring for bleeding—which may mitigate other risks and complicate the interpretation of the ulcer diagnosis itself. We have strengthened this discussion by citing relevant literature on the complexities of pharmacoepidemiology in cirrhosis.

**Comment 14: Platelet do not substantially contribute to INR and INR vs bleeding risk is very controversial association.**

**Response 14:** We sincerely thank the reviewer for highlighting the complex relationship between INR, platelet count, and bleeding risk, and for urging greater precision in our discussion. We agree that the contribution of platelets to INR is minimal, and that the association between INR and bleeding risk is multifactorial and context-dependent.

In direct response to this comment, we have removed any prior implication that platelets substantially influence INR values. Furthermore, we have revised the relevant section in our Discussion (Page 31) to provide a more nuanced and accurate interpretation. We now emphasize that INR primarily reflects impaired hepatic synthesis of coagulation factors and is a well-established marker of liver dysfunction and prognosis. We also explicitly acknowledge the controversy surrounding INR as a direct predictor of bleeding risk, citing relevant literature that illustrates how this relationship can be influenced by etiology, anticoagulant use, and other factors. Importantly, we strengthen our argument by referencing our own subgroup analyses, which demonstrated that INR remained a robust predictor of mortality irrespective of anticoagulation status, thereby supporting its value in risk stratification.

- **Responses to Reviewer #2:**

**Comment 1：Predictor Selection Process. The manuscript does not clearly describe how the final set of eight predictors was selected. It remains unclear whether the process was guided by data-driven techniques (e.g., penalized regression), clinical reasoning, or a hybrid approach. Recommendation: Please provide a detailed description of the predictor selection procedure, and indicate whether multicollinearity assessment or sensitivity analyses were conducted to ensure model robustness.**

**Response1:** We sincerely thank the reviewer for this important comment regarding the clarity of our predictor selection process. We agree that a more detailed description of the variable selection methodology was necessary. In response, we have revised the “Statistical Analysis” or “Methods” section to provide a step-by-step explanation of our procedure. Briefly, the initial candidate variables were first filtered based on clinical relevance and existing literature. Subsequently, univariate and multivariate logistic regression analyses were applied. Variables that remained significant were further refined using least absolute shrinkage and selection operator (LASSO) regression, a data-driven technique that effectively handles multicollinearity and prevents overfitting by penalizing regression coefficients. This hybrid approach—combining clinical knowledge with statistical regularization—resulted in the selection of eight non-redundant predictors with both statistical stability and clinical interpretability. The process of variable selection via LASSO is visually summarized in Fig 5.

Fig 5. Variable selection with LASSO. (A) LASSO cross-validation error curve. The number of variables selected at the optimal lambda value is 8. (B) LASSO coefficient plot. Variables with non-zero coefficients (marked "Selected: Yes") are retained, identifying key variables.

**Comment 2：Lack of ACLF-Specific Comparators. While MELD and OASIS scores are included for benchmarking, these scores are suboptimal for risk stratification in critically ill patients with acutely decompensated cirrhosis. Notably, the manuscript omits comparison with more appropriate tools such as the CLIF-OF score and ACLF-specific prognostic models, which have been developed and validated for precisely this clinical population. Recommendation: Please justify the exclusion of these scores and, where feasible, consider including them in the comparative analysis.**

**Response 2:** We sincerely thank the reviewer for this critical and insightful suggestion. We fully agree that comparison with ACLF-specific prognostic scores is essential for validating the clinical relevance and performance of our model in the target population of critically ill cirrhotic patients. In direct response to this recommendation, we have now included comparisons with the CLIF-OF and CLIF-C ACLF scores throughout our revised analysis. As presented on page 22 of the revised manuscript under "Discrimination Performance," our proposed model demonstrated superior discriminative ability for predicting short-term mortality when compared to these established ACLF-specific tools. Specifically, in the derivation cohort, the AUC of our model exceeded that of CLIF-OF (AUC 0.817) and CLIF-C ACLF (AUC 0.831) by 0.034 and 0.020, respectively. Similarly, in the validation cohort, our model outperformed both CLIF-OF (AUC 0.813) and CLIF-C ACLF (AUC 0.814), further supporting its robustness and potential clinical utility in this high-risk population(Fig 6).

Fig 6. Nical utility and predictive performance evaluation of the short-term mortality model in severe cirrhosis. (A) Receiver operating characteristic (ROC) curve comparisons of multiple predictive models in the derivation cohort. The proposed model (red curve) achieved superior discriminative performance (AUC = 0.846) compared to conventional scoring systems. (B) External validation of model performance in an independent cohort, demonstrating maintained predictive accuracy AUC = 0.843. (C) Decision curve analysis (DCA) in the derivation cohort showing enhanced net benefit of the proposed model (blue curve) across clinically relevant threshold probabilities (20–80%), outperforming existing models. (D) Validation cohort DCA confirming consistent clinical utility of the novel model (blue curve) compared to standard approaches.

**Comment 3： Inclusion of Patients with Malignancy.Inclusion of patients with metastatic malignancies may significantly influence the mortality signal in the model, as their risk profiles are likely dominated by non-hepatic factors. Most established prognostic models in cirrhosis exclude patients with active cancer.Question: How many patients with metastatic malignancies were included? We suggest conducting a sensitivity analysis excluding this subgroup, or at minimum, discussing the potential impact on model calibration and discrimination.**

**Response 3:** We sincerely thank the reviewer for this insightful comment regarding the inclusion of patients with malignancies. We fully agree that the prognosis of such patients is considerably more complex and likely dominated by non-hepatic factors, which may not align with the objective of developing a liver-specific prognostic model.

In our original cohort, 29 patients with metastatic malignancies were included. In direct response to this comment, we have excluded these patients from the primary analysis. To maintain cohort size and statistical power, we identified additional patients with decompensated cirrhosis without any malignancies from the same database who met all inclusion criteria. The model was subsequently retrained using this refined dataset, which enhances the clinical relevance and validity of our predictors for liver-related outcomes.

**Comment 4：Interpretation of Bilirubin and INR as Liver Function Markers**

**The use of total bilirubin and INR as direct proxies for hepatic synthetic function is potentially problematic. Total bilirubin may be elevated due to hemolysis or cholestasis, and INR can be substantially affected by anticoagulant use, sepsis, or vitamin K deficiency — factors that are common in this patient population. Recommendation: The limitations of using INR and bilirubin as liver-specific markers should be explicitly discussed. Please indicate whether anticoagulant use was assessed and whether this could have confounded the results.**

**Response 4:** We sincerely thank the reviewer for raising these critical points regarding the interpretation of bilirubin and INR as markers of liver function. We fully agree that both parameters can be influenced by multiple extrahepatic factors, and their interpretation requires careful clinical contextualization.

In response to this comment, we have substantially revised our discussion of bilirubin in the manuscript. We now provide a more nuanced explanation of the pathophysiological mechanisms underlying hyperbilirubinemia in cirrhosis, highlighting the significant contribution of hemolysis secondary to portal hypertension—including hypersplenism, altered erythrocyte membrane properties, and microcirculatory disturbances—in addition to impaired hepatic clearance and conjugation capacity. This expanded discussion, supported by appropriate references, offers a more comprehensive interpretation of total bilirubin levels beyond synthetic function alone.

Regarding INR, we have explicitly addressed the potential confounding effect of anticoagulant therapy. We extracted additional data on anticoagulant use at ICU admission and performed subgroup analyses comparing patients with and without anticoagulation. The results consistently demonstrated that INR remained a strong and independent predictor of short-term mortality in both subgroups, and its prognostic value was not materially altered by anticoagulant use. These findings, which we have now included in the Results section and visualized in Fig 7, support the robustness of INR as a prognostic variable in our model despite potential confounding factors.

Fig 7. Kaplan-Meier Analysis by Anticoagulant Use. (A) Anticoagulant Group: Short-term survival in severe cirrhosis stratified by international normalized ratio (INR) of prothrombin.(B) Non-Anticoagulant Group: Short-term survival in severe cirrhosis stratified by international normalized ratio (INR) of prothrombin.

**Comment 5：Limited Generalizability Beyond ICU Settings.As the cohort is derived exclusively from ICU patients, the generalizability of the model to other care settings (e.g., hepatology wards, general medicine) remains uncertain.Question: Do the authors intend to validate this model in non-ICU settings? If so, please provide further context or planned approaches.**

**Response 5:** We sincerely thank the reviewer for this insightful comment regarding the generalizability of our model beyond the ICU setting. We fully agree that our model, derived from a critically ill population, requires further validation in non-ICU environments such as hepatology wards or general medical units to establish its broader applicability. We have added a paragraph in the "Future Directions" section of our revised manuscript explicitly stating our plans to externally validate this model in non-ICU patient cohorts. We outline our intention to collaborate with tertiary hospitals possessing well-characterized cohorts of cirrhotic patients managed in ward settings, and to assess the model's calibration and discrimination in these populations. We also discuss the potential need for model refinement or reweighting of predictors should performance vary across care settings.

**Comment 6：Practical Implementation and Availability of the Tool.While the nomogram is a helpful visual representation, the manuscript does not provide any accessible clinical tool (e.g., calculator, software script, web application) for real-world use. Recommendation: Please clarify whether an open-access implementation of the model is planned or already in development, and whether it will be made publicly available.**

**Response 6:** We sincerely appreciate the reviewer’s valuable feedback regarding the practical application of our nomogram. In response to this suggestion, we are pleased to confirm that an open-access web-based calculator for the proposed model is currently under development. This tool will allow clinicians to input individual patient variables and easily obtain estimated probabilities, thereby facilitating routine clinical decision-making. We plan to host the calculator on a publicly available platform and will provide the source code to enhance reproducibility and allow for further validation. We intend to include a link to the tool in the final version of the manuscript upon its completion. This idea has been incorporated into the Future Directions section of our paper.

**Comment minor comments 1：Peptic Ulcer as a Protective Variable: The observed association between peptic ulcer disease and lower mortality likely reflects treatment-related confounding (e.g., PPI use). Causal inference should be avoided, and this result interpreted cautiously.**

**Response minor comments 1:** Thank you for this important comment. We agree that the observed association between peptic ulcer disease and lower mortality should not be interpreted causally, and that unmeasured confounding—particularly related to treatments such as proton pump inhibitors—may be driving this result. In the Discussion section (page 30 of the manuscript), we have already addressed this possibility by emphasizing that patients with peptic ulcers likely received more comprehensive pharmacological and supportive management, which may have contributed to the observed protective effect. We also explicitly stated that differences in baseline disease severity between groups may play a role. We will further clarify in the revision that this association requires cautious interpretation and is hypothesis-generating rather than demonstrative of a causal relationship.

**Comment minor comments 2：Use of Max/Min Lab Values: The rationale for using maximum or minimum lab values during hospitalization is understandable. However, a brief discussion on why admission values or averages were not used would be useful for the reader.**

**Response minor comments 2:** A brief discussion of this point has been included in the "Methodological Innovations" section of the Discussion “the use of extreme-value laboratory analysis to capture critical physiological states more accurately than averaged measures; the range between maximum and minimum values may cause the mean to inaccurately reflect the patient’s true clinical status. To mitigate this bias, we employed extremes analysis, which more reliably captures disease severity during hospitalization.”

**Comment minor comments 3：Language and Clarity: The manuscript is generally readable, but some sections are overly complex or dense. A language review would enhance clarity and readability.**

**Response minor comments 3:** Thank you for this suggestion. We have thoroughly revised the manuscript with a focus on improving language clarity and readability. This included simplifying complex sentence structures, streamlining the narrative flow, and ensuring consistent use of terminology. We believe these edits have significantly enhanced the overall quality and accessibility of the text.

**Comment minor comments 4：Structure of the Limitations Section: Consider organizing the limitations into thematic subheadings (e.g., data limitations, model assumptions, generalizability) for improved structure.**

**Response minor comments 4:** We appreciate this constructive suggestion. In response, we have reorganized the Limitations section under the following thematic subheadings: “Data Source and Generalizability”, “Data Handling and Missing Values”, and “Methodological Approach”. This new structure improves clarity and helps readers better contextualize and interpret the discussed limitations.
