# Supplementary material for: Predicting short-term mortality in severe cirrhosis: An interpretable machine learning model integrating routine clinical indicators
Source: PLoS One. 2026 Mar 3;21(3):e0328952. doi: 10.1371/journal.pone.0328952 (PMC12956121; doi:10.1371/journal.pone.0328952)
Supplement: Attachment — Submitted filename: Response_to_Reviewers_auresp_2.docx [file pone.0328952.s010.docx]

**Response to Reviewers**

**Manuscript ID:** PONE-D-25-36378R1

**Title:** Predicting Short-Term Mortality in Severe Cirrhosis: An Interpretable Machine Learning Model Integrating Routine Clinical Indicators

**Dear Dr. Sona Frankova and Reviewers,**

We are deeply grateful to you and the reviewers for the insightful feedback provided during the review of our manuscript. Your valuable comments have significantly enhanced the scientific rigor and overall quality of our work. In response, we have thoroughly considered all suggestions and have diligently addressed each point raised by the reviewers in our point-by-point response. For your convenience, all corresponding modifications in the manuscript have been clearly highlighted in the "Revised Manuscript with Track Changes" file.

- **Response to editor**

**Comment 1: Please include the following items when submitting your revised manuscript:**

**A rebuttal letter that responds to each point raised by the academic editor and reviewer(s). You should upload this letter as a separate file labeled 'Response to Reviewers'. A marked-up copy of your manuscript that highlights changes made to the original version. You should upload this as a separate file labeled 'Revised Manuscript with Track Changes'. An unmarked version of your revised paper without tracked changes. You should upload this as a separate file labeled 'Manuscript'.**

**Response 1:** We appreciate the opportunity to submit a revised manuscript. We have carefully addressed all editor and reviewer feedback. In accordance with the guidelines, we are including our point-by-point response, a manuscript with highlighted changes, and an unmarked version.

**Comment 2: If you would like to make changes to your financial disclosure, please include your updated statement in your cover letter. Guidelines for resubmitting your figure files are available below the reviewer comments at the end of this letter.**

**Response 2:** Thank you for your suggestions. Our Financial Disclosure Statement remains unchanged at this time. Besides, we have carefully addressed all points raised in the figure guidelines, we utilized NAAS, PLOS's free image analysis tool, to process our images in order to meet publication standards and each image has been revised accordingly. The specific changes are reflected in the updated figure files.

**Comment 3: If applicable, we recommend that you deposit your laboratory protocols in protocols.io to enhance the reproducibility of your results. Protocols.io assigns your protocol its own identifier (DOI) so that it can be cited independently in the future. For instructions see: https://journals.plos.org/plosone/s/submission-guidelines#loc-laboratory-protocols. Additionally, PLOS ONE offers an option for publishing peer-reviewed Lab Protocol articles, which describe protocols hosted on protocols.io. Read more information on sharing protocols at** [**https://plos.org/protocols?utm_medium=editorial-email&utm_source=authorletters&utm_campaign=protocols.**](https://plos.org/protocols?utm_medium=editorial-email&utm_source=authorletters&utm_campaign=protocols.)

**Response 3:** We thank you for your advice. In accordance with your guidance, the laboratory protocol for this study has been deposited on protocols.io, where it has been assigned a citable Digital Object Identifier (DOI), provided below: DOI:10.17605/OSF.IO/FYNCG.

**Comment 4: If the reviewer comments include a recommendation to cite specific previously published works, please review and evaluate these publications to determine whether they are relevant and should be cited. There is no requirement to cite these works unless the editor has indicated otherwise.**

**Response 4:** Thank you for highlighting this important aspect of manuscript preparation. In our detailed point-by-point response to the reviewers, we have addressed all concerns raised. In this process, we noted that there were no specific directives from the reviewers concerning the addition of citations.

**Comment 5: Please review your reference list to ensure that it is complete and correct. If you have cited papers that have been retracted, please include the rationale for doing so in the manuscript text, or remove these references and replace them with relevant current references. Any changes to the reference list should be mentioned in the rebuttal letter that accompanies your revised manuscript. If you need to cite a retracted article, indicate the article’s retracted status in the References list and also include a citation and full reference for the retraction notice.**

**Response 5:** Thank you for your guidance. Following a thorough verification, we confirm that all references in our manuscript have been accurately sequenced and are correct. Furthermore, we have ensured that none of the cited articles have been retracted.

- **Responses to Reviewer #2:**

**Comment 1: External Validation. The model remains internally validated only. The authors now recognize this limitation and have described their plans for external validation, which is appreciated. However, the manuscript should emphasize even more clearly that the model is preliminary and not yet suitable for generalization beyond the MIMIC-IV ICU population. A short clarification in both the Abstract and Conclusions would be sufficient.**

**Response 1:** Thank you very much for your valuable feedback. Our model has not yet undergone external validation, which will be an important part of our future research. Following your suggestion, we have clarified in both the Abstract and Conclusion sections that the model is currently not suitable for application outside the ICU population. You may refer to the Conclusion section of the Abstract in the manuscript, which states:

Conclusions: Our prediction model identifies high-risk cirrhotic patients and highlights critical prognostic factors, offering clinicians a valuable tool for risk stratification and timely intervention. The strong correlation between laboratory markers, complications, and outcomes underscores the importance of close monitoring in this population. However, our model is an initial step, effective within the ICU but requiring external, multi-center studies to broaden its clinical applicability, which is a clear priority for our future work.

**Comment 2: Use of Extreme Laboratory Values. The justification for using maximum or minimum laboratory values has been provided, but this approach may introduce bias by incorporating information unavailable at admission. A brief note in the Limitations or Supplementary Material confirming that model performance was stable when admission values were tested would further support the robustness of the analysis.**

**Response 2:** We sincerely thank you for this insightful comment. In response, we have performed an additional sensitivity analysis of our model. We recognized that employing maximum or minimum laboratory values in practical applications might introduce bias by incorporating information not available at the time of admission. To address this, we re-extracted the first available laboratory measurements after admission and incorporated these initial values into the model for validation. The results showed that the model using the initial laboratory values achieved an AUC of 0.771 in the training set and 0.767 in the validation set. Although these values are slightly lower than those of the model utilizing extreme values (training AUC = 0.837; validation AUC = 0.825), they remain well above 0.5 (Fig. 1). This indicates that the initial laboratory values still enable our model to predict short-term mortality effectively, thereby mitigating potential bias from missing admission-time data. Moreover, the model remains applicable for mortality prediction in patients with only a single laboratory measurement. We have elaborated on this aspect in the "Methodological Innovations" section of the manuscript as follows:

**Methodological Innovations**

This study is distinguished by three key advancements: the systematic integration of comorbidities, addressing oversights in previous models; the use of extreme-value laboratory analysis to capture critical physiological states more accurately than averaged measures; the range between maximum and minimum values may cause the mean to inaccurately reflect the patient's true clinical status. To mitigate this bias, we employed extreme analysis, which more reliably captures disease severity during hospitalization, although the use of extreme values may introduce bias by incorporating post-admission information. A post-hoc sensitivity analysis demonstrated stable predictive performance when only initial admission values were used (Training AUC: 0.771; Validation AUC: 0.767), thereby supporting the robustness of the model (S5 Fig); and the introduction of clinically actionable INR stratification, replacing traditional binary classifications. These innovations yielded a model demonstrating superior discrimination compared to existing scores while maintaining practicality through the use of routine parameters.

**Fig 1. Sensitivity analysis of the prediction model.** (A) ROC curves of the model in the training cohort. The blue curve represents the model using extreme laboratory values (AUC = 0.837), and the red curve represents the model using the first recorded values at admission (AUC = 0.771). (B) ROC curves of the model in the validation cohort. The blue curve corresponds to the model using extreme laboratory values (AUC = 0.825), and the red curve corresponds to the model using the first recorded values at admission (AUC = 0.767).

**Comment 3: Scope and Discussion. The expanded discussion now provides a thoughtful pathophysiological explanation of bilirubin, INR, and hemolysis, which is appreciated. However, some sections remain somewhat repetitive or overly detailed. A more concise summary that focuses on the clinical implications of the findings would improve readability and impact.**

**Response 3:** We sincerely thank you for this valuable suggestion. We agree that the previous description in this section was overly detailed and did not sufficiently highlight our key findings. Accordingly, we have thoroughly revised the relevant paragraph on page 26 of the manuscript. The updated text now reads:

Total bilirubin emerged as a significant predictor of short-term mortality in severe cirrhosis, a finding supported by its odds ratio (OR 1.027, 95% CI 1.002–1.052) in our model and consistent prognostic value in survival analysis (HR 1.023, 95% CI 1.010–1.037, p = 0.001). The elevation in total bilirubin reflects both hepatic and extrahepatic pathophysiological processes. On one hand, portal hypertension-induced hypersplenism promotes chronic hemolysis through splenic sequestration and destruction of red blood cells, exacerbated by altered hemorheological properties and microvascular stress. This leads to increased indirect bilirubin from hemoglobin breakdown. On the other hand, intrahepatic cholestasis and impaired hepatocyte function reduce bilirubin clearance, elevating direct bilirubin levels. Additionally, in decompensated cirrhosis, diminished glucuronidation capacity further contributes to the accumulation of indirect bilirubin. Together, these mechanisms underpin the strong association between elevated total bilirubin and mortality, highlighting its utility in risk stratification and early intervention for high-risk cirrhotic patients.

- **Response to Minor Comments**

**Comment 1: The English language has improved substantially, although a brief professional language check before publication would help ensure clarity and consistency throughout.**

**Response 1:** Thank you for this suggestion. We have thoroughly reviewed the language throughout the manuscript and have revised several statements to ensure greater scientific clarity and precision.

**Comment 2: Figures are clear and informative; however, a few could be slightly simplified for publication.**

**Response 2:** We thank the reviewer for this suggestion. We agree that the original figures had some limitations. Accordingly, all figures have been revised to ensure they meet the standards for publication in terms of clarity and presentation.

**Comment 3: If possible, please include a reference or note to the planned web-based calculator (e.g., "to be available at...") before final submission.**

**Response 3:** We sincerely thank you for this suggestion. In response, we have developed a preliminary web-based calculator for our prediction model, which is now accessible at: <https://9zhangshun.shinyapps.io/livercirrhosis/.> Additionally, we have added a corresponding statement in the "Future Directions" section of the manuscript to inform readers of this resource.
